# Global Distribution of Mycobacterium tuberculosis Spoligotypes

**DOI:** 10.3201/eid0811.020125

**Published:** 2002-11

**Authors:** Ingrid Filliol, Jeffrey R. Driscoll, Dick van Soolingen, Barry N. Kreiswirth, Kristin Kremer, Georges Valétudie, Dang Duc Anh, Rachael Barlow, Dilip Banerjee, Pablo J. Bifani, Karin Brudey, Angel Cataldi, Robert C. Cooksey, Debby V. Cousins, Jeremy W. Dale, Odir A. Dellagostin, Francis Drobniewski, Guido Engelmann, Séverine Ferdinand, Deborah Gascoyne-Binzi, Max Gordon, M. Cristina Gutierrez, Walter H. Haas, Herre Heersma, Gunilla Källenius, Eric Kassa-Kelembho, Tuija Koivula, Ho Minh Ly, Athanasios Makristathis, Caterina Mammina, Gerald Martin, Peter Moström, Igor Mokrousov, Valérie Narbonne, Olga Narvskaya, Antonino Nastasi, Sara Ngo Niobe-Eyangoh, Jean W Pape, Voahangy Rasolofo-Razanamparany, Malin Ridell, M. Lucia Rossetti, Fritz Stauffer, Philip N. Suffys, Howard Takiff, Jeanne Texier-Maugein, Véronique Vincent, Jacobus H. de Waard, Christophe Sola, Nalin Rastogi

**Affiliations:** * Institut Pasteur, Pointe-à-Pitre, Guadeloupe, French West Indies; † Wadsworth Center, Albany, New York, USA; ‡ National Institute of Public Health and the Environment, Bilthoven, the Netherlands; § Public Health Research Institute, New York, New York, USA; ¶ National Institute of Hygiene and Epidemiology, Hanoi, Vietnam; # General Infirmary, Leeds, U.K.; ** St. Georges’ Hospital Medical School, London, U.K.; †† Instituto de Biotecnologia, Castelar, Argentina; ‡‡ Centers for Disease Control and Prevention, Atlanta, Georgia, USA; §§ Australian Reference Laboratory for Bovine Tuberculosis, Department of Agriculture, South Perth, Australia; ¶¶ University of Surrey, Guildford, Surrey, U.K.; ## Universidade Federal, Pelotas, Brazil; *** Public Health Laboratory Service, Dulwich Hospital, London, U.K.; ††† University Children’s Hospital, Heidelberg, Germany; ‡‡‡ Institut Pasteur, Paris, France; §§§ Robert Koch Institute, Berlin, Germany; ¶¶¶ Swedish Institute for Infectious Disease Control, Solna, Sweden; ### Institut Pasteur, Bangui, Central African Republic; **** Hygiene-Institut der Universität, Wien, Austria; †††† University of Palermo, Palermo, Italy; ‡‡‡‡ Bundesinstitut für gesundheitlichenVerbraucherschutz und Veterinärmedizin, Jena, Germany; §§§§ Pasteur Institute of Saint Petersburg, Saint Petersburg, Russia; ¶¶¶¶ Centre Hospitalier Universitaire, Brest, France; #### University of Firenze, Firenze, Italy; ***** Les Centres Gheskio, Institut National de Laboratoire et de Recherche, Port-au- Prince, Haïti; ††††† Cornell University, Ithaca, New York, USA; ‡‡‡‡‡ Institut Pasteur, Tananarive, Madagascar; §§§§§ Göteborg University, Göteborg, Sweden; ¶¶¶¶¶ Universidade Federal do Rio Grande do Sul, Brazil; ##### Bundesstaatliche bakteriologisch-serologische Untersuchungsanstalt Wien, Austria; ****** Oswaldo Cruz Institute, Rio de Janeiro, Brazil; †††††† Caracas**,** Venezuela; ‡‡‡‡‡‡ Centre Hospitalier Universitaire, Bordeaux, France; §§§§§§ Instituto de Investigacionas Cientificas, Caracas, Venezuela

**Keywords:** Mycobacterium tuberculosis, spoligotyping

## Abstract

We present a short summary of recent observations on the global distribution of the major clades of the *Mycobacterium tuberculosis* complex, the causative agent of tuberculosis. This global distribution was defined by data-mining of an international spoligotyping database, SpolDB3. This database contains 11,708 patterns from as many clinical isolates originating from more than 90 countries. The 11,708 spoligotypes were clustered into 813 shared types. A total of 1,300 orphan patterns (clinical isolates showing a unique spoligotype) were also detected.

Since the publication of the second version of our spoligotypes database on *Mycobacterium tuberculosis* (1), the causative agent of tuberculosis (TB), the proportion of clustered isolates (shared types [STs]) increased from 84% (2,779/3,319) to 90% (11,708/13,008). Fifty percent of the clustered isolates were found in only 20 STs. Three of these isolates are *M. bovis,* including *M. bovis* BCG (ST 481, 482, and 683). The addition of the next 30 most frequent STs increased the total proportion of clustered isolates (65% instead of 50% initially).

A total of 36 potential subfamilies or subclades of *M. tuberculosis* complex have been tentatively identified, leading to the definition of major and minor visual recognition rules (Table). The ancestral East-African Indian family (EAI) is made up of at least five main subclades, whereas at least three major spoligotyping patterns are found within the Haarlem family (2). Two families found in central and Middle Eastern Asia (CAS1 and CAS2) are newly defined. The X family (3) is also currently split into at least three well-defined subclades. However, the subdivision of family T (T1–T4, likely to represent relatively old genotypes), which differs from the classic ST 53 (all spacers present except 33–36), remains poorly defined. Similarly, the Latino-American and Mediterranean family (LAM) is tentatively split into subclades LAM1–LAM10 (4). Spoligotyping used alone is not well suited for studying the phylogeny of these two clades (T and LAM). Such study will require results from other genotyping methods such as IS*6110*-restriction fragment length polymorphism (5) or mycobacterial interspersed repetitive units–variable number of DNA tandem repeats (6). Among well-characterized major clades of tubercle bacilli, four families represent 35% of 11,708 clustered isolates (Beijing 11%, LAM 9.3%, Haarlem 7.5%, and the X clade 7%).

**Table:** Excerpt from SpolDB3 database showing prototype spoligotypes, visual recognition rules, and binary and octal description^a^

Rk	ST	Class^b^	Total (n)^c^	Rules^d^	Binary description	Octal
1	1	Beijing	1282	∆1–34	οοοοοοοοοοοοοοοοοοοοοοοοοοοοοοοοοοννννννννν	000000000003771
2	53	T1	864	F	ννννννννννννννννννννννννννννννννοοοοννννννν	777777777760771
11	52	T2	163	∆40 and F	ννννννννννννννννννννννννννννννννοοοονννοννν	777777777760731
30	37	T3	71	∆13 and F	ννννννννννννονννννννννννννννννννοοοοννννννν	777737777760771
64	40	T4	26	∆19 and F	ννννννννννννννννννονννννννννννννοοοοννννννν	777777377760771
7	47	Haarlem1	246	∆26–30 and E	νννννννννννννννννννννννννοοοοοονοοοοννννννν	777777774020771
20	2	Haarlem2	104	∆1–24, ∆26–30 and E	οοοοοοοοοοοοοοοοοοοοοοοονοοοοοονοοοοννννννν	000000004020771
3	50	Haarlem3	519	E	ννννννννννννννννννννννννννννννονοοοοννννννν	777777777720771
6	119	X1	310	C	νννννννννννννννννοννννννννννννννοοοοννννννν	777776777760771
4	137	X2	427	C and ∆39–42	νννννννννννννννννοννννννννννννννοοοοννοοοον	777776777760601
31	92	X3	70	∆4–12 and C	νννοοοοοοοοονννννοννννννννννννννοοοοννννννν	700036777760731
15	48	EAI1	118	A and ∆40	ννννννννννννννννννννννννννννοοοονονννννοννν	777777777413731
13	19	EAI2	130	∆3, ∆20–21 and A	ννοννννννννννννννννοονννννννοοοονοννννννννν	677777477413771
16	11	EAI3	121	∆2-3, A and ∆37–39	νοονννννννννννννννννννννννννοοοονοννοοονννν	477777777413071
8	139	EAI4	234	∆26–27 and A	νννννννννννννννννννννννννοονοοοονοννννννννν	777777774413771
46	236	EAI5	41	A	ννννννννννννννννννννννννννννοοοονοννννννννν	777777777413771
24	181	Afri1	91	∆7–9 and ∆39	ννννννοοονννννννννννννννννννννννννννννονννν	770777777777671
ND	331	Afri2	9	∆8–12, ∆21–24 and ∆37–39	νννννννοοοοοννννννννοοοοννννννννννννοοονννν	774077607777071
ND	438	Afri3	3	∆8–12 and ∆37–39	νννννννοοοοοννννννννννννννννννννννννοοονννν	774077777777071
17	482	*M. bovis*-BCG	26	∆3, ∆9, ∆16 and D	ννονννννοννννννοννννννννννννννννννννννοοοοο	676773777777600
ND	641	*M. microti*	8	4-7, 23–24, 37–38	οοοννννοοοοοοοοοοοοοοοννοοοοοοοοοοοοννοοοοο	074000030000600
ND	592	*M. canetti*	6	30 and 36	οοοοοοοοοοοοοοοοοοοοοοοοοοοοονοοοοονοοοοοοο	000000000101000
21	26	CAS1	102	∆4–7, ∆23–34	νννοοοονννννννννννννννοοοοοοοοοοοοννννννννν	703777740003771
ND	288	CAS2	6	∆4–10, ∆23–34	νννοοοοοοοννννννννννννοοοοοοοοοοοοννννννννν	700377740003771
12	20	LAM1	152	∆3 and B	ννονννννννννννννννννοοοοννννννννοοοοννννννν	677777607760771
22	17	LAM2	92	∆3, ∆13 and B	ννονννννννννονννννννοοοοννννννννοοοοννννννν	677737607760771
19	33	LAM3	108	∆9–11 and B	ννννννννοοονννννννννοοοοννννννννοοοοννννννν	776177607760771
49	60	LAM4	37	∆40 and B	ννννννννννννννννννννοοοοννννννννοοοονννοννν	777777607760731
42	93	LAM5	44	∆13 and B	ννννννννννννονννννννοοοοννννννννοοοοννννννν	777737607760771
37	64	LAM6	47	∆29 and B	ννννννννννννννννννννοοοοννννονννοοοοννννννν	777777607560771
36	41	LAM7	48	∆20, ∆26-27 and B	νννννννννννννννννννοοοοονοονννννοοοοννννννν	777777404760771
NA	290	LAM8	9	∆27 and B	ννννννννννννννννννννοοοοννονννννοοοοννννννν	777777606760771
5	42	LAM9^e^	344	B	ννννννννννννννννννννοοοοννννννννοοοοννννννν	777777607760771
9	61	LAM10	202	∆23–25 and F	ννννννννννννννννννννννοοονννννννοοοοννννννν	777777743760771
26	34	S^f^	82	∆9–10 and F	ννννννννοοννννννννννννννννννννννοοοοννννννν	776377777760771
28	451	H37Rv	78	∆20–21 and F	νννννννννννννννννννοονννννννννννοοοοννννννν	777777477760771

^a^Rk, ranking no.; ND, not done; ST, arbitrary designation; *M.,* Mycobacterium. ^b^Class: family definition. See text for the definition of the family acronyms. ^c^Total (n), size of the class; binary and octal, description. ^d^Rule A, absence of spacers 29–32, presence of spacer 33 and absence of spacer 34; rule B, absence of spacers 21–24 and spacers 33–36; rule C : absence of spacer 18 and spacers 33–36; rule D, absence of spacers 39–43; rule E, absence of spacer 31 and spacers 3–-36; rule F, absence of spacers 33–36. Clades defined with low sample size, such as Afri2, Afri3, CAS2, and LAM8 are subject to change. ^e^Formerly LAM1. ^f^Formerly LAM2.

The global distribution of the most frequently observed spoligotypes by continent in SpolDB3 is as follows. Among the patterns originating in North America (n= 4,276, 32% of the total number of isolates in the database), 16% of the strains are of the Beijing type, 14% belong to ST 137 or ST 119 (X family), and 8% are unique (results not shown). In Central America (n=587, 4.5%), 8% of the strains belong to the ubiquitous ST 53, 7% are ST 50, and 6% are ST 2; the last two STs are part of the Haarlem family. In South America (n=861, 6.6%), the distribution of ST 53 and ST 50 accounts for 10% and 9%, respectively, of the spoligotypes, whereas ST 42 accounts for as much as 9% of the total isolates. The origin of ST 42 remains to be established. In Africa (n=1,432, 11%), ST 59 and ST 53 account for 9% of all isolates studied thus far; however, the values obtained for ST 59 are biased because strains from Zimbabwe are overrepresented. We also observed that *M. africanum* ST 181 accounts for as much as 6% of all spoligotypes from Africa in our sample.

In Europe (n=4,360, 33.5%), ST 53 represents as much as 9% of the spoligotypes, ST 50 and 47 (Haarlem family) represent 8% of the cases, and the Beijing family accounts for 4% of the spoligotypes. In the Middle Eastern and central Asian region, where the number of samples obtained is still very low (n=351, 2.7%), a high diversity of strains within the EAI and CAS families has been observed, and no single pattern currently exceeds 5%. Further studies of isolates from these regions are needed, e.g., in India, where our sampling is still anecdotal (n=44 isolates). Notwithstanding the scarcity of available data from this region, the observed diversity suggests that this region might be of great interest for further study of the genetic variation of tubercle bacilli. Contrary to what we observed for the Middle East and central Asia, the Far East Asian region (n=801, 6.1%) is characterized by the prevalence of a single genotype, the Beijing type family, a family linked to emerging multiresistance (7). One out of two strains in the Far East is a Beijing type. In Oceania (n=340, 2.6%), ST 19 and Beijing account for 15% and 13%, respectively, of clustered isolates. Thus, this preliminary analysis of the spoligotype distribution of SpolDB3 clearly shows major differences in the population structure of tubercle bacilli within the eight subcontinents studied (Africa; Europe; North America; Central America; South America; Middle East and Central Asia; Far East Asia; and Oceania).

At present, SpolDB 3 is an experimental tool that has yet to prove its usefulness in tracking epidemics. Nevertheless, the facility with which matches between spoligotypes can be detected suggests that this tool may be a good screening mechanism for population-based studies on recent TB transmission. Indeed, the detection of a rarely found ST in SpolDB3 may be a catalyst that signals researchers to look for the clonality of the isolates and to study their epidemiologic relatedness.

Data-exchange protocols through inter-networking will also be implemented in the near future. Working groups such as the European Network for Exchange of Molecular Typing Information (available from: URL: www.rivm.nl/enemti) are coordinating such initiatives. The expanded use of the Bionumerics software (third upgrade; Applied Maths, St. Martens-Latem, Belgium) may also foster this research field. SpolDB3 will also be instrumental in facilitating better understanding of the driving forces that shape tubercle bacilli evolution. Further research should now emphasize the use of data-mining methods, in combination with experts’ knowledge, to tackle the complex dynamics of the population's genetics of tubercle bacilli and TB transmission (3). Our sample represents the compilation of many national studies and, as such, should be considered as an ongoing population-based project aimed at studying global TB genetic diversity. Nevertheless, obtaining a more precise and representative snapshot of the genetic variability of *M. tuberculosis* complex will require a larger sampling. Although only partially representative of worldwide spoligotypes of *M. tuberculosis* complex, Spo1DB3 contains a reservoir of genetic information that has already proved useful for defining the phylogenetic links that exist within the TB genomes and for constructing theoretical models of genome evolution. Much remains to be done to evaluate the potential of global genetic databases to better characterize casual contacts (that could lead to identification of sporadic cases) in TB epidemiology. An improved version of our database, which will focus on areas with a high prevalence of TB, is currently in development; as of August 26, 2002, it had 20,000 isolates and 3,000 alleles. Ongoing population-based genotyping projects will likely help shed light on contemporary and ancient tubercle bacilli’s evolutionary history.

## References

[1] Sola C, Filliol I, Guttierez CM, Mokrousov I, Vincent V, Rastogi N. Spoligotype database of *Mycobacterium tuberculosis*: biogeographical distribution of shared types and epidemiologic and phylogenetic perspectives. Emerg Infect Dis. 2001;7:390–6. 11384514 10.3201/eid0703.010304 PMC2631784

[2] Kremer K, van Soolingen D, Frothingham R, Haas WH, Hermans PWM, Martin C, Comparison of methods based on different molecular epidemiological markers for typing of *Mycobacterium tuberculosis* strains: interlaboratory study of discriminatory power and reproducibility. J Clin Microbiol. 1999;37:2607–18. 10405410 10.1128/jcm.37.8.2607-2618.1999 PMC85295

[3] Sebban M, Mokrousov I, Rastogi N, Sola C. A data-mining approach to spacer oligonucleotide typing of *Mycobacterium tuberculosis.* Bioinformatics. 2002;18:235–43. 10.1093/bioinformatics/18.2.235 11847071

[4] Sola C, Filliol I, Legrand E, Mokrousov I, Rastogi N. *Mycobacterium tuberculosis* phylogeny reconstruction based on combined numerical analysis with IS*1081*, IS*6110*, VNTR and DR-based spoligotyping suggests the existence of two new phylogeographical clades. J Mol Evol. 2001;53:680–9. 10.1007/s002390010255 11677628

[5] van Embden JDA, Cave MD, Crawford JT, Dale JW, Eisenach KD, Gicquel B, Strain identification of *Mycobacterium tuberculosis* by DNA fingerprinting: recommendations for a standardized methodology. J Clin Microbiol. 1993;31:406–9. 8381814 10.1128/jcm.31.2.406-409.1993 PMC262774

[6] Supply P, Lesjean S, Savine E, Kremer K, van Soolingen D, Locht C. Automated high-throughput genotyping for the study of global epidemiology of *Mycobacterium tuberculosis* based on mycobacterial interspersed repetitive units. J Clin Microbiol. 2001;39:3563–71. 10.1128/JCM.39.10.3563-3571.2001 11574573 PMC88389

[7] Glynn JR, Whiteley J, Bifani PJ, Kremer K, van Soolingen D. Worldwide occurrence of Beijing/W strains of *Mycobacterium tuberculosis*: a systematic review. Emerg Infect Dis. 2002;8:843–9. 12141971 10.3201/eid0808.020002 PMC2732522

